# Determining optimal cutoff scores of Cognitive Abilities Screening Instrument to identify dementia and mild cognitive impairment in Taiwan

**DOI:** 10.1186/s12877-024-04810-y

**Published:** 2024-03-02

**Authors:** Wan-Jing Lyu, Pai-Yi Chiu, Chung-Hsiang Liu, Yu-Chi Liao, Hsin-Te Chang

**Affiliations:** 1 Department of Psychology, College of Medical and Health Sciences, Asia University, Taichung, Taiwan; 2 Department of Neurology, Show Chwan Memorial Hospital, Changhua City, Changhua, Taiwan; 3 Department of Neurology, College of Medicine, China Medical University Hospital, China Medical University, Taichung, Taiwan; 4 Research Assistance Center, Show Chwan Memorial Hospital, Changhua City, Changhua, Taiwan; 5 Department of Psychology, College of Science, Chung Yuan Christian University, No. 200, Zhongbei Road, Taoyuan 320, Taiwan

**Keywords:** Aging, Cognitive assessment, Cognitive disorders, Dementia, Memory

## Abstract

**Background:**

The early detection of dementia depends on efficient methods for the assessment of cognitive capacity. Existing cognitive screening tools are ill-suited to the differentiation of cognitive status, particularly when dealing with early-stage impairment.

**Methods:**

The study included 8,979 individuals (> 50 years) with unimpaired cognitive functions, mild cognitive impairment (MCI), or dementia. This study sought to determine optimal cutoffs values for the Cognitive Abilities Screening Instrument (CASI) aimed at differentiating between individuals with or without dementia as well as between individuals with or without mild cognitive impairment. Cox proportional hazards models were used to evaluate the value of CASI tasks in predicting conversion from MCI to all-cause dementia, dementia of Alzheimer’s type (DAT), or to vascular dementia (VaD).

**Results:**

Our optimized cutoff scores achieved high accuracy in differentiating between individuals with or without dementia (AUC = 0.87—0.93) and moderate accuracy in differentiating between CU and MCI individuals (AUC = 0.67 – 0.74). Among individuals without cognitive impairment, scores that were at least 1.5 × the standard deviation below the mean scores on CASI memory tasks were predictive of conversion to dementia within roughly 2 years after the first assessment (all-cause dementia: hazard ratio [HR] = 2.81 – 3.53; DAT: 1.28 – 1.49; VaD: 1.58). Note that the cutoff scores derived in this study were lower than those reported in previous studies.

**Conclusion:**

Our results in this study underline the importance of establishing optimal cutoff scores for individuals with specific demographic characteristics and establishing profiles by which to guide CASI analysis.

**Supplementary Information:**

The online version contains supplementary material available at 10.1186/s12877-024-04810-y.

## Introduction

Determining the cognitive status of elderly individuals can be exceedingly difficult when dealing with early-stage dementia [1]. Cognitive assessments play a key role in the diagnosis of dementia and its prodromal stage, mild cognitive impairment (MCI) [2, 3]; however, comprehensive neuropsychological assessments are time-consuming and not universally applicable [3, 4]. Cognitive screening tests (CSTs) are a cost-effective alternative to assessing cognitive function. The Cognitive Abilities Screening Instrument (CASI) has been widely used CST in research; however, its low sensitivity leads to a high false negative rate [3] in the early detection of dementia [5, 6].

When dealing with dementia or MCI, detection sensitivity could be affected by any number of factors [7, 8], including inter- and intra-study variations in demographic characteristics [4]. Researchers have listed education level and age as important demographic variable capable of affecting cognitive test scores [9, 10]. Accurate determinations of cognitive status depend on optimal cutoff scores for individuals with specific demographic characteristics. Furthermore, most CSTs rely on global scores, despite the fact that individuals with MCI are prone to cognitive impairment in specific cognitive domains rather than in general cognitive functioning [2, 11].

In the current study, we sought to derive optimal cutoff scores for CASI among a large sample of Taiwanese individuals (*n* = 8,979). We also sought to derive cutoff scores for screening tests used to predict MCI or dementia. Finally, we derived cutoff scores for various CASI subtests based on the mean score and standard deviation. This study was based on three hypotheses: (1) CASI scores can be used to differentiate individuals according to cognitive status as unimpaired, MCI, and dementia; and (2) CASI scores can be used to predict the conversion of unimpaired individuals to MCI or dementia.

## Methods

### Participants

**Fig. 1:**
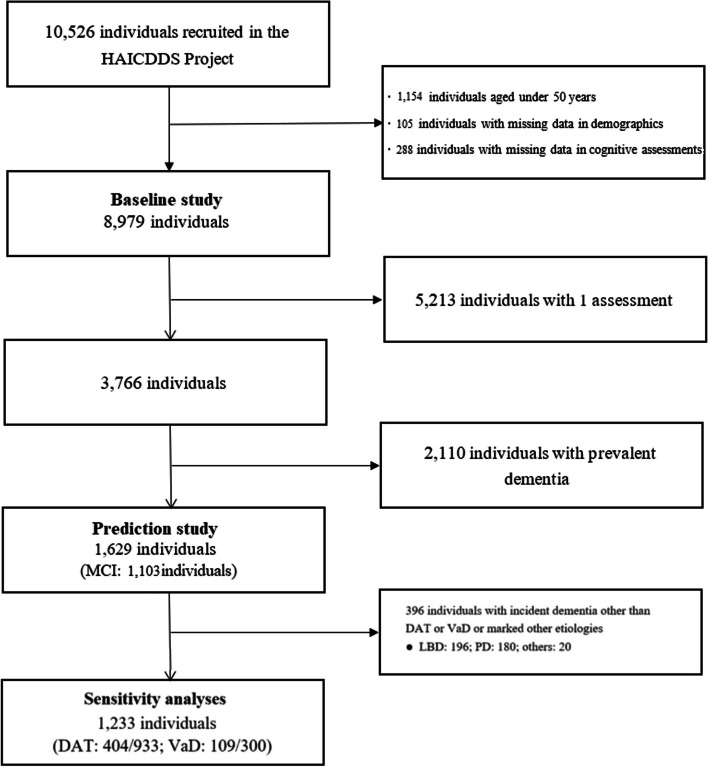
Selection process of study subjects. DAT: Dementia of Alzheimer’s type. HAICDDS: History-Based Artificial Intelligent Clinical Dementia Diagnostic System. LBD: Lewy body dementia. *MCI* Mild cognitive impairment. *PD* Parkinson’s disease. *VaD* Vascular dementia

Study subjects were selected from a longitudinal dementia registry dataset established for the History-based Artificial Intelligent Clinical Dementia Diagnostic System (HAICDDS) by the Show Chwan Healthcare System, which covers all of Taiwan except the eastern region [12–16]. The objective in establishing HAICDDS was to enable regular examinations (serum tests, cognitive assessment tests, and laboratory examinations) to facilitate the early detection and prevention of dementia. At present, the dataset includes 10,526 participants, comprising 20,018 data points. We selected 8,979 individuals over 50 years of age who completed CASI (Fig. 1). Among this cohort, 1,629 individuals were followed for an average of 2 $\pm$ 1.05 years (range: 0.5 – 6 years) to assess the predictive value of the respective cognitive task scores on conversion to dementia. These individuals were diagnosed with incident dementia of Alzheimer’s type (DAT) or vascular dementia (VaD) during follow-up sessions in accordance with criteria proposed by the National Institute on Aging and Alzheimer’s Association (NIA-AA) [17], the National Institute of Neurological Disorders and Stroke (NINDS), and the Association Internationale pour la Recherche et l'Enseignement en Neurosciences (AIREN) [18, 19]. Computed tomography (CT) scans or magnetic resonance imaging (MRI) scans captured using a Siemens 3 T scanner were used to aid in the diagnosis and excluding apparent etiologies other than degenerative or cerebrovascular conditions. MCI patients and CU individuals without incident dementia were categorized into AD or vascular origin groups based on the clinical diagnosis criteria put forth by previous studies [20, 21] or by retrospectively excluding other etiologies, respectively.

Note that the subjects excluded from the prediction study tended to be older (77.56 $\pm$ 8.59 years), male (56.70%), and possessing a lower education level (4.30 $\pm$ 4.12 years; *p* < 0.05). Global cognitive function among participants was assessed by clinical neuropsychologists using CASI [22] and the Montreal Cognitive Assessment (MoCA) [23, 24]. Note also that this study used cutoff scores suggested by Lin et al. (2002) for the determination of cognitive deficits. The activities of daily living (ADL) were assessed using the History-based Artificial Intelligent ADL questionnaire (HAI-ADL) [25]. A diagnosis of cognitively unimpaired (CU) required a global Clinical Dementia Rating (CDR)-sum of boxes (CDR-SB) score of 0 or 0.5 [26, 27] and normally performance on CASI. Individuals with a CDR-SB score of 0.5 were assigned to the CU group, due to the fact that most individuals with a score of this level maintain cognitive stability [27]. The inclusion of a clinical sample in this study suggests that some of the subtle changes indicative of cognitive decline may not have been captured by the tests in this study. MCI was diagnosed in accordance with criteria proposed in previous studies [28], and an operational determination of MCI was indicated by a CASI score below the cutoff score, a normal performance on HAI-ADL, and a CDR score of at least 0.5. A diagnosis of dementia was based on NIA-AA criteria [17], and an operational determination of impaired cognitive function or ADL was indicated by performance below the cutoffs for CASI and HAI-ADL [22]. The study was approved by the Institutional Review Board of the Show Chwan Memorial Hospital (SCMH_IRB1081006). Informed consent was waived because of the retrospective nature of the study and the analysis used anonymous clinical data.

#### CASI

CASI comprises 25 tasks selected from the Hasegawa Dementia Screening Scale, the Mini-Mental State Examination, and the Modified Mini-Mental State Test [11, 25]. The CASI assesses nine cognitive domains, including long-term memory, short-term memory, attention, mental manipulation, orientation, abstract thinking, language, visuospatial construction, and verbal fluency. Long-term memory was assessed in terms of general knowledge (e.g., “mooncakes are typical desserts during the celebration of which holiday?”) (max score = 10). Short-term memory was assessed by having the participant repeat three words read aloud by the examiner (max score = 12). Attention was assessed by having the participant repeat two syntactically complex sentences (max score = 8). Mental manipulation was assessed by asking participants to subtract 7 from 100 consecutively 5 times and then repeat digits read as read aloud by the examiner or in reverse order (max score = 10). Orientation was assessed by asking the participants about their current location, date, and time (max score = 18). Abstract thinking was assessed by having participants describe the similarities between two objects (e.g., a shrimp and a fish; a table and a chair) (max score = 12). Language skills were assessed by having participants write five common Chinese characters and perform confrontational naming of body parts and common objects (max score = 10). Visuospatial construction was assessed by having the participant copy two partially overlapping pentagons (max score = 10). Verbal fluency was assessed by having the participants name as many four-legged animals as they could in a 30-s period (max score = 10). Note that in the short-term memory task, three points were awarded if the participant spontaneously recalled each of the target words, two points were awarded if the participant recalled the target words after category cueing (e.g., “something to wear” for hat), and one point was awarded if the participant recalled the target words after the examiner provided three choices (e.g., “shoes, hat, socks”).

In a previous study, Lin et al. (2002) established cut-off scores to differentiate between individuals with dementia and those deemed cognitively healthy in a group of 2,096 elderly Taiwanese. The cognitively healthy individuals were recruited from Kinmen County and the patients with dementia were recruited from Taipei. The cut-off scores were as follows: illiterate individuals (49/50), individuals with an education below the elementary level (67/68); and individuals with an elementary level education or higher (79/80). These scores resulted in sensitivity of 0.83–0.89 and specificity of 0.85–0.91.

### Statistical analysis

We compared demographic and clinical characteristics among CU individuals and those with MCI or dementia using multiple one-way analysis of variance (ANOVA) or chi-square tests. We sought to establish optimal cutoffs for four specific demographic subgroups stratified according to the demographical distributions of the sample and Taiwanese population [29]: younger individuals (< 75 years) with a relatively low education level (< 6 years of education; *n* = 698), younger individuals with higher education level ($\ge$ 6 years of education; *n* = 3,068), older individuals ($\ge$ 75 years) with a relatively low education level (*n* = 3,015) and older individuals with higher education level (*n* = 2,198). Further a priori multiple regression analysis revealed that gender did not have a significant effect on CASI scores (*p* = 0.30 – 0.91). Independent sample t-tests and chi-square tests were used to analyze demographic and clinical characteristics. Receiver operating characteristic (ROC) curves were used to assess the performances of CASI cut-off scores in differentiating among participants in terms of cognitive status. CASI scores compared individuals with and without dementia as well as individuals with or without mild cognitive impairment (MCI). Optimal cut-off scores were derived using the Youden Index as follows: maximum = sensitivity + specificity – 1. In deriving the cutoff score for suspected cognitive impairment, we adopted a conventional criterion suggested in previous studies; i.e., 1.5 × the standard deviation below the reference or normative mean on the total score and the score for each of the cognitive domains [28, 30]. In the longitudinally predictive study, we adopted the criterion for determination of predictive values of scores below the cutoff on conversion to dementia. The distribution of scores in the CU group was used as a reference. Cox proportional hazard regression models were used to compare the risk of incident dementia as a function of global score or the scores for the various cognitive domains, after controlling for the effects of demographic variables. Schoenfeld residuals were examined using visual scanning to assess whether the proportional hazard (PH) assumption for Cox regression was violated [31]. The random scattering of residuals indicated that the PH assumption was not violated.

## Results

### Demographics and clinical characteristics

Table 1 presents the demographic and clinical characteristics in each group. The average age was higher in the dementia group than in the CU and MCI groups, in the following subsamples: total (*F*_2, 8976_ = 959.778, *p* < .001), older (illiterate: *F*_2, 2161_ = 78.48, *p* < .001; older-low education: *F*_2, 3014_ = 87.817, *p* < .001; older-high education: *F*_2, 2197_ = 109.615, *p* < .001), and younger/ high education subsamples (*F*_2, 3067_ = 22.209, *p* < .001). The average age of subjects was higher in the MCI group than in the CU group, including the total sample and younger-high education and older-high education subsamples. The proportion of females in the dementia group was higher in the CU and MCI groups: total sample (*χ*^2^_*df* = 2,* n* = 8979_ = 46.69, *p* < .001). The proportion of males was higher in all younger subsample groups (more educated: *χ*^2^_*df* = 2, *n* = 3068_ = 11.89, *p* = 0.003; less educated sample: *χ*^2^_*df* = 2, *n* = 313_ = 6.23, *p* = 0.044), except the illiterate subsample (*p* = 0.047, no difference in post-hoc comparison). The education level was higher in the CU group than in the MCI and dementia groups in the following subsamples: total (*F*_2, 8976_ = 533.49, *p* < .001) and younger/ high education (*F*_2, 3067_ = 49.42, *p* < .001). In the older-high education subsample, the mean education level was higher in the CU group than in the dementia group (*F*_2, 2197_ = 3.26, *p* = 0.04).

**Table 1:** Demographical and clinical characteristics of participants

	**CU**	**MCI**	**Dementia**	**Statistical comparison**
*Total n* = *8,979*				
N	1914	2337	4728	
Age (years, mean [SD])	67.84 (10.49)^ab^	74.89 (10.95)^ac^	78.99 (9.60)^bc^	*F*_2, 8976_ = 959.79, *p* < 0.001
Gender (% female [n])	50.00 (957)^ab^	53.66 (1254)^ac^	58.74 (2777)^bc^	*χ*^2^_*df* = 2,* n* = 8979_ = 46.69, *p* < 0.001
Education (years, mean [SD])	8.12 (4.71)^ab^	5.55 (4.79)^ac^	4.22 (4.40)^bc^	*F*_2, 8976_ = 533.49, *p* < 0.001
CDR-SB (mean [SD])	0.27 (0.28)^ab^	1.70 (0.77)^ac^	9.15 (4.71)^bc^	*F*_2, 8976_ = 6290.83, *p* < 0.001
*Younger-illiterate n* = *385*				
N	75	120	190	
Age (years, mean [SD])	68.32 (5.65)	69.59 (4.57)	69.43 (4.41)	*F*_2, 384_ = 1.91, *p* = 0.150
Gender (% female [n])	92.0 (69)	92.5 (111)	84.21 (160)	*χ*^2^_*df* = 2, *n* = 385_ = 6.12, *p* = 0.047
Education (years, mean [SD])	0 (–)	0 (–)	0 (–)	
CDR-SB (mean [SD])	0.27 (0.26)^ab^	1.63 (0.73)^ac^	8.31 (4.29)^bc^	*F*_2, 384_ = 273.43, *p* < 0.001
*Younger-low education n* = *313*				
N	73	114	126	
Age (years, mean [SD])	68.21 (4.10)	68.29 (4.24)	68.22 (4.99)	*F*_2, 312_ = 0.01, *p* = 0.990
Gender (% female [n])	68.49 (50)	71.93 (82)^c^	57.14 (72)^c^	*χ*^2^_*df* = 2, *n* = 313_ = 6.23, *p* = 0.044
Education (years, mean [SD])	3.12 (1.19)	3.10 (1.32)	2.94 (1.24)	*F*_2, 312_ = 0.64, *p* = 0.529
CDR-SB (mean [SD])	0.33 (0.28)^ab^	1.66 (0.68)^ac^	8.08 (4.38)^bc^	*F*_2, 312_ = 233.90, *p* < 0.001
*Younger-high education n* = *3,068*				
N	1215	985	868	
Age (years, mean [SD])	62.37 (8.25)^ab^	63.66 (7.88)^ac^	64.72 (7.91)^bc^	*F*_2, 3067_ = 22.21, *p* < 0.001
Gender (% female [n])	47.16 (573)^b^	46.70 (460)^c^	40.09 (348)^bc^	*χ*^2^_*df* = 2, *n* = 3068_ = 11.89, *p* = 0.003
Education (years, mean [SD])	10.01 (3.65)^ab^	9.07 (3.34)^ac^	8.56 (3.08)^bc^	*F*_2, 3067_ = 49.42, *p* < 0.001
CDR-SB (mean [SD])	0.24 (0.28)^ab^	1.60 (0.74)^ac^	8.05 (4.53)^bc^	*F*_2, 3067_ = 276.80, *p* < 0.001
*Older-illiterate n* = *2162*				
N	135	386	1641	
Age (years, mean [SD])	80.31 (4.33)^b^	80.61 (3.87)^c^	83.69 (5.30)^bc^	*F*_2, 2161_ = 78.48, *p* < 0.001
Gender (% female [n])	76.30 (103)	79.02 (305)	80.80 (1326)	*χ*^2^_*df* = 2, *n* = 2162_ = 2.01, *p* = 0.365
Education (years, mean [SD])	0 (–)	0 (–)	0 (–)	
CDR-SB (mean [SD])	0.30 (0.27)^ab^	1.89 (0.85)^ac^	10.16 (4.81)^bc^	*F*_2, 2161_ = 848.92, *p* < 0.001
*Older-low education n* = *853*				
N	70	175	608	
Age (years, mean [SD])	80.69 (4.48)^b^	81.53 (3.87)^c^	82.82 (4.48)^bc^	*F*_2, 852_ = 11.69, *p* < 0.001
Gender (% female [n])	48.57 (34)	52.57 (92)	58.39 (355)	*χ*^2^_*df* = 2, *n* =853_ = 3.77 *p* = 0.152
Education (years, mean [SD])	2.73 (1.15)	2.86 (1.29)	2.76 (1.29)	*F*_2, 852_ = 0.42, *p* = 0.657
CDR-SB (mean [SD])	0.34 (0.28)^ab^	1.81 (0.82)^ac^	9.07 (4.64)^bc^	*F*_2, 852_ = 335.11, *p* < 0.001
*Older-high education n* = *2,198*				
N	346	557	1295	
Age (years, mean [SD])	79.39 (3.86)^ab^	80.57 (4.55)^ac^	83.25 (5.46)^bc^	*F*_2, 2197_ = 109.62, *p* < 0.001
Gender (% female [n])	36.99 (128)	36.62 (204)	39.85 (516)	*χ*^2^_*df* = 2, *n* = 2198_ = 2.14, *p* = 0.343
Education (years, mean [SD])	8.57 (3.37)^b^	8.23 (3.16)	8.08 (3.10)^b^	*F*_2, 2197_ = 3.26, *p* = 0.039
CDR-SB (mean [SD])	0.31 (0.29)^ab^	1.72 (0.76)^ac^	8.87 (4.56)^bc^	*F*_2, 2197_ = 1287.07, *p* < 0.001

Younger: < 75 years; Older: ≥ 75 years; Low education: < 6 years; High education: ≥ 6 years; ^*a*^ CU ≠ MCI; ^*b*^ CU ≠ Dementia; ^*c*^ MCI ≠ Dementia; All *p* values < 0.001. *Abbreviations.* *CU* Cognitively unimpaired, *MCI* Mild cognitive impairment, *SD* Standard deviation, *CDR-SB* Clinical Dementia Rating-sum of boxes

Among the CU individuals who underwent follow-up assessments (n = 526), 25.5% converted to dementia (*n* = 134). Among the MCI individuals who underwent follow-up assessments (*n* = 1,103), 39.7% converted to dementia (*n* = 438). Note that the follow-up duration of MCI converters was higher than that of MCI non-converters. Among MCI converters, the proportion of females was higher (*χ*^2^_*df* = 1, *n* =1,103_ = 4.76, *p* = 0.03), the education level was lower (*t* = 4.31, *p* < 0.001), the average age was higher (*t* = 5.13, *p* < 0.001), and scores on CDR-Sum of Boxes were higher (*t* = 4.00, *p* < 0.001). In the CU group, we observed no significant demographics differences between converters and non-converters (*p* = 0.06 – 0.58) (Supplementary Table 1).

### Differentiating cognitive status based on total CASI scores

#### Dementia vs. no dementia

Table 2 lists the effectiveness of CASI in discriminating between individuals with or without dementia and MCI vs. CU. In discriminating between individuals with or without dementia, the CASI cut-off scores optimized for age were as follows: ≤ 75 years (72/73: AUC = 0.92, sensitivity = 0.83, specificity = 0.84, *p* < 0.001) and > 75 years (55/56: AUC = 0.89, sensitivity = 0.82, specificity = 0.80, *p* < 0.001). The CASI cut-off scores optimized for education level were as follows: illiterate (47/48: AUC = 0.90, sensitivity = 0.84, specificity = 0.81, *p* < 0.001), ≤ 6 years of education (55/56: AUC = 0.88, sensitivity = 0.84, specificity = 0.77, *p* < 0.001), and > 6 years of education (72/73: AUC = 0.93, sensitivity = 0.84, specificity = 0.86, *p* < 0.001). The CASI cut-off scores optimized for younger subjects were as follows: younger-illiterate (50/51: AUC = 0.89, sensitivity = 0.88, specificity = 0.76, *p* < 0.001), younger-low education (60/61: AUC = 0.87, sensitivity = 0.83, specificity = 0.91, *p* < 0.001), and younger-high education (76/77: AUC = 0.92, sensitivity = 0.84, specificity = 0.86, *p* < 0.001). The CASI cut-off scores optimized for older subjects were as follows: older-illiterate (42/43: AUC = 0.90, sensitivity = 0.88, specificity = 0.75, *p* < 0.001), older-low education (53/54: AUC = 0.87, sensitivity = 0.85, specificity = 0.75, *p* < 0.001), and older-high education (63/64: AUC = 0.91, sensitivity = 0.87, specificity = 0.78, *p* < 0.001).

**Table 2:** AUC and cutoff points of CASI

Group	AUC	95% CI	Cutoff	Sensitivity	Specificity
*Dementia vs. No dementia*					
Age (year)					
< 75	0.92	0.91–0.92	72/73	0.83	0.84
≥ 75	0.89	0.89–0.90	55/56	0.82	0.80
Education (year)					
0	0.90	0.89–0.92	47/48	0.84	0.81
< 6	0.88	0.87–0.90	55/56	0.84	0.77
≥ 6	0.93	0.92–0.94	72/73	0.84	0.86
Younger-illiterate	0.89	0.86–0.92	50/51	0.88	0.76
Younger-low education	0.87	0.83–0.91	60/61	0.83	0.76
Younger-high education	0.92	0.91–0.94	76/77	0.84	0.86
Older-illiterate	0.90	0.89–0.91	42/43	0.88	0.75
Older-low education	0.87	0.85–0.90	53/54	0.85	0.75
Older-high education	0.91	0.89–0.72	63/64	0.87	0.78
*CU vs. MCI*					
Age					
< 75	0.69	0.67–0.71	81/82	0.78	0.52
≥ 75	0.73	0.71–0.76	72/73	0.72	0.66
Education					
0	0.74	0.70–0.78	64/65	0.67	0.71
< 6	0.73	0.68–0.78	68/69	0.78	0.62
≥ 6	0.71	0.69–0.72	81/82	0.78	0.54
Younger-illiterate	0.72	0.65–0.80	64/65	0.81	0.56
Younger-low education	0.69	0.61–0.76	68/69	0.84	0.47
Younger-high education	0.67	0.65–0.70	83/84	0.78	0.48
Older-illiterate	0.73	0.68–0.78	62/63	0.67	0.68
Older-low education	0.75	0.68–0.82	67/68	0.74	0.69
Older-high education	0.73	0.70–0.77	77/78	0.76	0.62

Younger: < 75 years; Older: ≥ 75 years; Low education: < 6 years; High education: ≥ 6 years. All *p* values < .001. *Abbreviations.* *AUC* Area under the curve, *CASI* Cognitive assessment screening instrument, *CI* Confidence interval

#### CU vs. MCI

In discriminating individuals with CU or MCI, the CASI cut-off scores optimized for age were as follows: ≤ 75 years of age (81/82: AUC = 0.69, sensitivity = 0.78, specificity = 0.52, *p* < 0.001) and > 75 years (72/73: AUC = 0.73, sensitivity = 0.72, specificity = 0.66, *p* < 0.001). The CASI cut-off scores optimized for education level were as follows: illiterate (64/65: AUC = 0.74, sensitivity = 0.67, specificity = 0.71, *p* < 0.001), ≤ 6 years of education (68/69: AUC = 0.73, sensitivity = 0.78, specificity = 0.62, *p* < 0.001), and > 6 years of education (81/82: AUC = 0.71, sensitivity = 0.78, specificity = 0.54, *p* < 0.001). The CASI cut-off scores optimized for younger subjects were as follows: younger-illiterate (64/65; AUC = 0.72, sensitivity = 0.81, specificity = 0.56, *p* < 0.001), younger-low education (68/69: AUC = 0.69, sensitivity = 0.84, specificity = 0.47, *p* < 0.001), and younger high education (83/84: AUC = 0.67, sensitivity = 0.78, specificity = 0.48, *p* < 0.001). The CASI cut-off scores optimized for older subjects were as follows: older-illiterate (62/63: AUC = 0.73, sensitivity = 0.67, specificity = 0.68, *p* < 0.001), older-low education (67/68: AUC = 0.75, sensitivity = 0.74, specificity = 0.69, *p* < 0.001), and older-high education (77/78: AUC = 0.73, sensitivity = 0.76, specificity = 0.62, *p* < 0.001).

### Risk of conversion to dementia

**Fig. 2:**
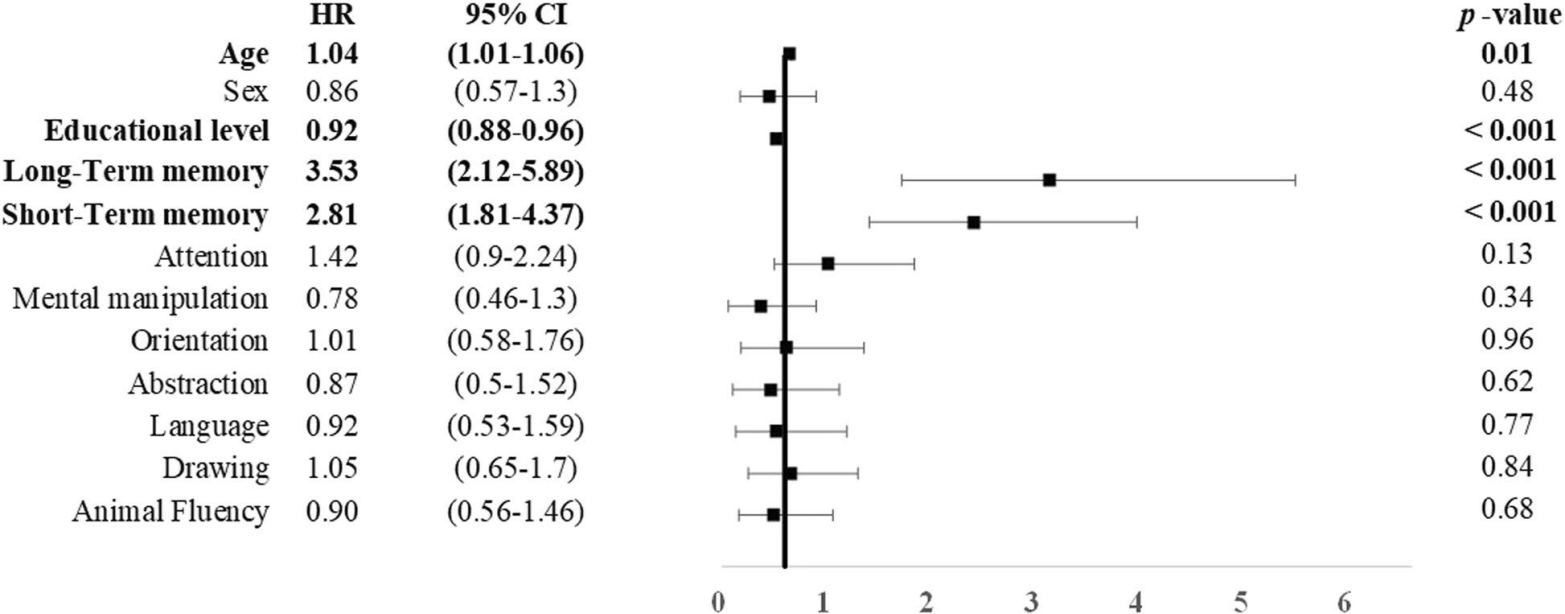
Risk of incident dementia among the participants. Poor performance on each task of CASI was defined as performance -1.5 × the SD below the mean of the CU group. *CI* Confidence interval, *HR* Hazard ratio. Bold font indicates statistically significant

Table 3 lists the CASI cutoff scores derived for each of the cognitive domains based on the degree of dispersion beyond 1.5 × the SD of mean scores in the CU group. After controlling for the effects of demographics, we determined that performance values below the cutoff scores for the long-term memory subtest (*B* = 1.26, hazard ratio [HR] = 3.53, CI = 2.12 – 5.89) and short-term memory subtest (*B* = 1.03, HR = 2.81, CI = 1.81 – 4.37) were predictive of conversion to dementia. None of the other subtests were predictive of conversion (*p* = 0.13 – 0.96) (Fig. 2 and Supplementary Fig. 1).

**Table 3:** The cutoff scores for CASI subscales

Subscale	Mean (SD)	Approximately 1.5 SD score
Long-term Memory (maximum score = 10)	9.71 (0.85)	8
Short-term Memory (maximum score = 12)	9.89 (2.13)	6
Attention (maximum score = 8)	6.32 (1.77)	3
Mental Manipulation (maximum score = 10)	6.99 (2.81)	2
Orientation (maximum score = 18)	16.87 (2.16)	13
Abstract Thinking (maximum score = 12)	6.48 (2.19)	3
Language (maximum score = 10)	9.23 (1.08)	7
Drawing (maximum score = 10)	9.11 (2.03)	6
Animal Fluency (maximum score = 10)	8.78 (1.81)	6

*Abbreviations.* *CASI* Cognitive assessment screening instrument (maximum: 100), *SD* Standard deviation

**Fig. 3:**
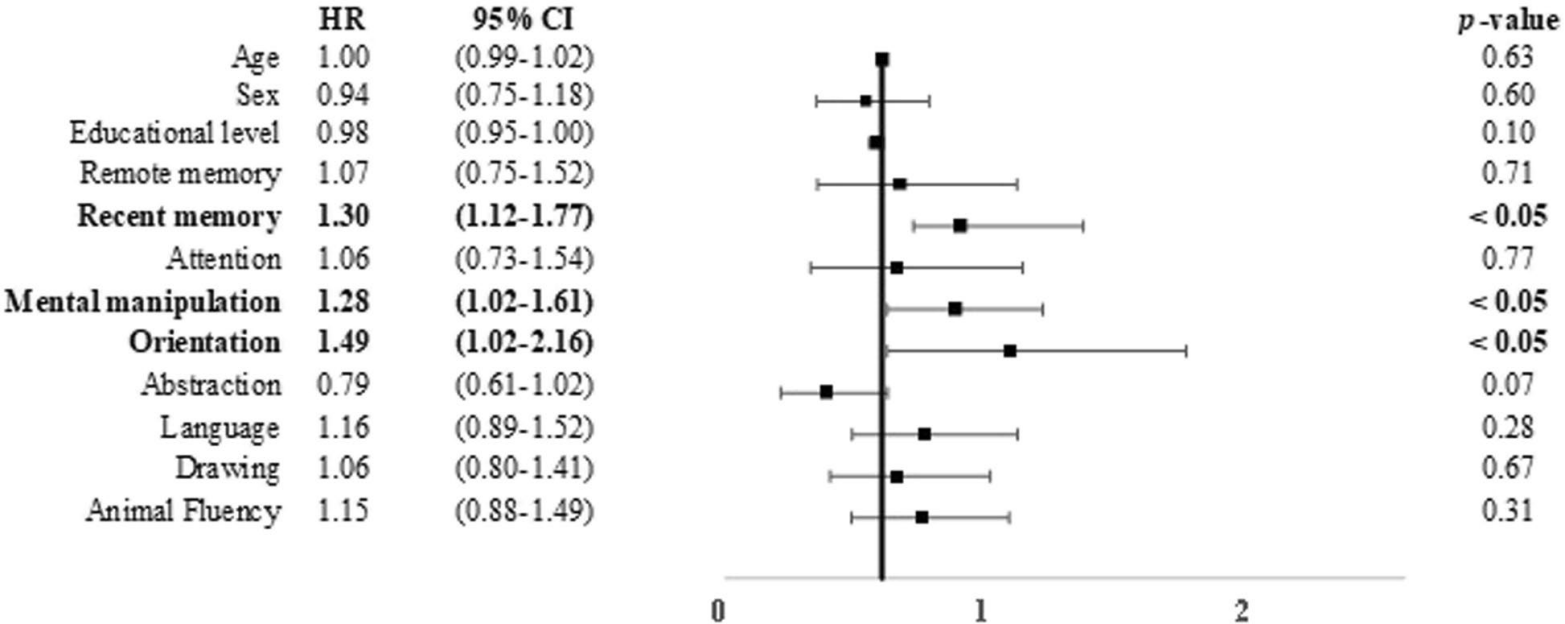
Risk of incident DAT among the participants. Notes are the same as those used in Fig. 2

**Fig. 4:**
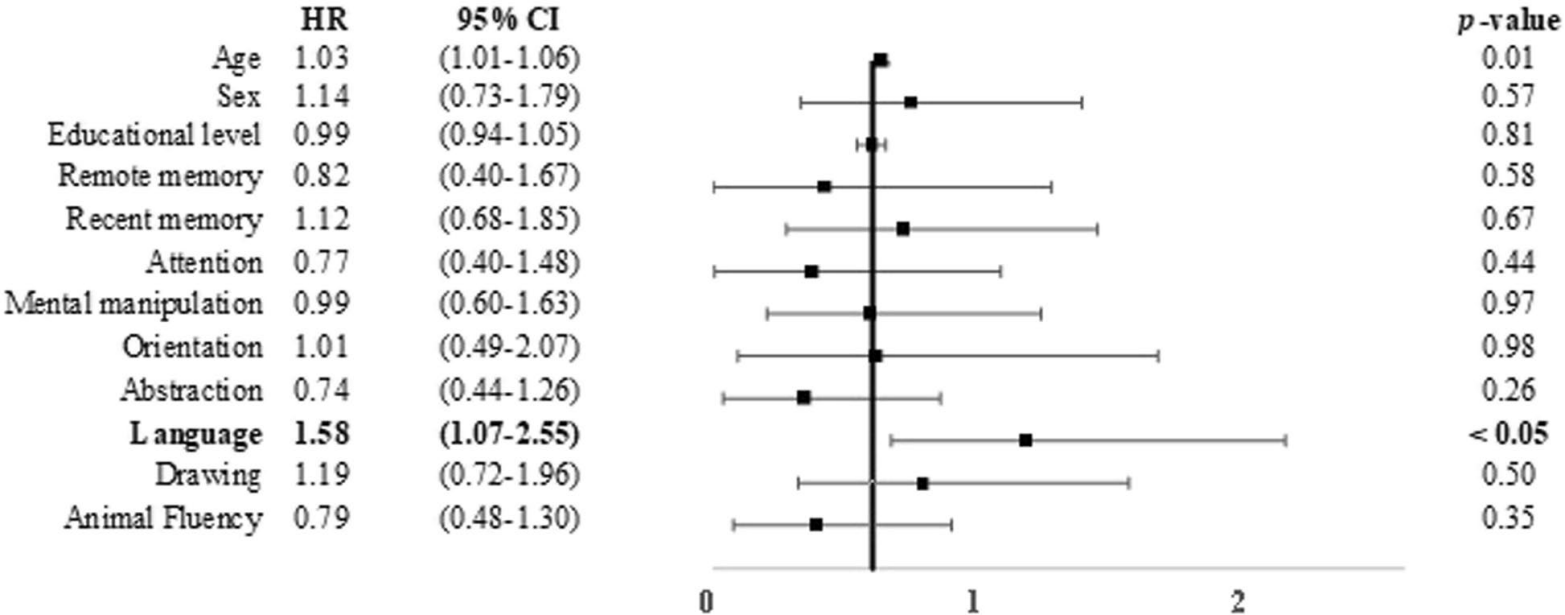
Risk of incident VaD among the participants. Notes are the same as those used in Fig. 2

Supplementary Table 2 lists the demographic and clinical characteristics of individuals with suspected AD or vascular etiologies. Compared to individuals with suspected AD, those with vascular etiologies were younger (*F*_1, 1231_ = 16.25, *p* < 0.001), better educated (*F*_1, 1231_ = 4.25, *p* < 0.05), and more likely to be male (*χ*^2^_*df* = 1,* n* = 1233_ = 39.38, *p* < 0.001). Individuals with vascular etiologies also displayed relatively poor performance on the CDR-SB during the first assessment (*F*_1, 1231_ = 6.20, *p* < 0.05). Scores below the cutoff scores for the short-term memory subtest (*B* = 0.41, HR = 1.30, CI = 1.12 – 1.77), mental manipulation (*B* = 0.35, HR = 1.28, CI = 1.02 – 1.61), and orientation (*B* = 0.40, HR = 1.49, CI = 1.02 – 2.16) were predictive of conversion to DAT (Fig. 3 and Supplementary Fig. 2). Scores below the cutoff score for the language subtest were predictive of conversion to VaD (*B* = 0.46, HR = 1.58, CI = 1.07 – 2.55) (Fig. 4 and Supplementary Fig. 3).

## Discussion

The study investigated the optimization of CASI cutoff scores with the aim of differentiating between individuals with or without dementia and in differentiating between individuals with MCI and those without cognitive impairment. Total CASI scores proved effective in differentiating between individuals with or without dementia across samples stratified according to demographic variables. Total CASI scores proved moderately effective in differentiating between MCI and CU individuals (lower specificity). After controlling for demographics, the CASI memory subtests were predictive of conversion from CU to dementia.

In line with previous studies [11, 22, 32], we determined that total CASI scores could be used to differentiate between individuals with or without dementia. Using the Mandarin Chinese version of CASI among Taiwanese individuals, Lin et al. [22] established optimal cutoff scores by which to differentiate between individuals with dementia and those with normal cognitive function. Note however that our cutoff scores for differentiating individuals with or without dementia were lower than those reported by Lin et al. [22] (illiterate: 49/50 vs. 47/48; low education: 67/68 vs. 55/56; high education: 79/80 vs. 72/73; younger- illiterate: 51/52 vs. 50/51; younger-low education: 67/68 vs. 60/61; younger- high education: 79/80 vs. 76/77; older-illiterate: 46/47 vs. 42/43; older-low education: 67/68 vs. 53/54; older-high education: 79/80 vs. 63/64). These differences may be attributed to our inclusion of patients with MCI in the non-dementia group, which may have enhanced the specificity of CASI scores in diagnosing dementia. Subsequent *post-hoc* sensitivity analysis in which patients with MCI were excluded from the non-dementia group revealed results that were very similar to those reported by Lin et al. [22] with the exception of higher cutoff scores for the illiterate groups in the current study (younger-illiterate: 58/59; older-illiterate: 50/51) (Supplementary Table 3).

Researchers have posited that MCI is a prodromal stage of dementia. MCI is generally defined as cognitive performance roughly 1.5 × SD below the normative mean in at least one cognitive domain with preservation of daily function. Teng et al. [11] suggested that sensitivity in detecting cognitive dysfunctions may differ among the various CASI tasks. They reported that short-term memory, orientation, and verbal fluency were the most effective indicators to differentiate dementia from normal cognitive function. To the best of our knowledge, no previous study has investigated the applicability of scores for CST sub-domains in detecting MCI or predicting conversion to dementia. In the current study, we determined that defining MCI as long-term memory and short-term memory scores 1.5 × the SD below the mean of the CU group was predictive of conversion to dementia. Amnestic MCI (aMCI) has been proposed as an indicator of conversion to dementia of Alzheimer’s type (DAT) [30, 33, 34]. Thus, our findings pertaining to the predictive value of CASI memory tasks may reflect a link between aMCI and related pathologically changes in the medial temporal lobe (MTL) and prefrontal cortex (PFC) and conversion to DAT [35, 36]. Note that in predicting conversion to dementia, the value of test scores for long-term memory exceeded those for short-term memory. This can perhaps be attributed to the fact that long-term memory tasks focus on long-term semantic memory retrieval (i.e., regularities and conventions), which may provide advantages in detecting early pathological changes in the MTL and PFC [37–39]. This may be explained by the fact that the retrieval of long-term semantic memory requires activation of the PFC [40], which interacts closely with the MTL. Interruptions to this interaction may be an early indicator of dementia.

Among the patients with incident DAT, performance on memory tasks and tasks assessing mental manipulation and orientation were also predictive of conversion to　dementia. This may be due to the fact that early changes in the interactions between the MTL and PFC were predictive of conversion to DAT [37–39, 41]. Note that performance on long-term memory tasks was predictive only of incident all-cause dementia. These results can perhaps be attributed to the fact that the retrieval of higher-order semantic knowledge is associated with lesions in the frontosubcortical and temporal polar regions. These regions may be compromised in patients with etiologies other than AD [42–44].

Among patients with VaD, performance on language tasks was predictive of conversion to dementia. This is perhaps associated with vulnerabilities in the blood vessels associated with core linguistic regions, which are supported by the middle cerebral artery (MCA), the most vulnerable artery in the brain in terms of stroke [45].

In the current study, the global CASI scores proved effective in differentiating between individuals with MCI and those without cognitive impairment; however, the discriminatory power (specificity) was low. The low specificity can perhaps be attributed to the fact that the education level of many of subjects in the current study was lower than in previous studies [11, 22, 32]. This implies that the lower total CASI scores reflect a lower educational level rather than pathological changes. Taken together, it appears that specificity in MCI detection and accuracy in predicting conversion to dementia might be better served using long-term and short-term memory tasks instead of global scores.

Our findings echo those in previous studies [e.g., [9, 46, 47] indicating that education could act as a protective factor against dementia. It is possible that education enhances cognitive reserve or that a higher education and the corresponding affluence permit a healthier lifestyle (e.g., access to healthy food and time for exercise) [47, 48].

This study established optimal CASI cutoff scores to facilitate differentiation between individuals with or without dementia as well as between individuals with CU or MCI. Our findings also shed light on the predictive value of the various CASI tasks and the mechanisms underlying dementia progression. However, this study was subject to various limitations. First, the subjects were recruited at neurology clinics, such that the study group was not selected at random (selection bias). Second, it is very likely that the individuals identified as CU did not in fact retain completely normal cognitive function, considering the fact that they were seeking help from clinicians. Thus, the applicability of these cutoff scores will require further confirmation in community-wide populations. Third, the relatively short follow-up duration in this study prevented an investigation into the predictive value of CASI scores across individuals with different etiologies. Fourth, the patients were categorized using screening cognitive tests, such that some of the individuals in the CU group may actually have displayed cognitive changes that were too subtle for detection using these tests [2].

This study established optimal cutoff scores by which to determine the cognitive status of elderly individuals visiting neurology clinics. The fact that the optimal cutoff scores were lower than those reported in previous studies indicates the need to formulate population-specific cutoff scores. In the current study, performance on CASI memory tasks was found to be predictive of conversion to dementia; however, further research with longer follow-up period will be required to assess the generalizability of our findings. In the future, it may also be possible to derive short-forms of CASI that focus on memory tasks.

### Supplementary Information

**Supplementary Material 1. **

## Data Availability

Data available on request due to privacy/ethical restrictions. Requests to access these datasets should be directed to H-T C, changht@cycu.edu.tw.
