# Supplementary material for: Determining optimal cutoff scores of Cognitive Abilities Screening Instrument to identify dementia and mild cognitive impairment in Taiwan
Source: BMC Geriatr. 2024 Mar 2;24:216. doi: 10.1186/s12877-024-04810-y (PMC10909252; doi:10.1186/s12877-024-04810-y)
Supplement: Supplementary file 1 — Supplementary Material 1. [file 12877_2024_4810_MOESM1_ESM.docx]

**Supplementary Table 1.** Demographic and clinical characteristics of converters and non-converters

|  | CU  (n = 526) | | MCI  (n = 1,103) | |
| --- | --- | --- | --- | --- |
|  | Non-converter | Converter | Non-converter | Converter |
| n (%) | 392 (74.52) | 134 (25.48) | 665 (58.85) | 438 (38.76) |
| Gender (% female)^b^ | 48.00 (188/392) | 50.70 (68/134) | 53.40 (355/665) | 60.00 (263/438) |
| Age (SD)^b^ | 69.83 (9.51) | 71.28 (9.16) | 72.44 (9.65) | 75.46 (9.45) |
| Education (SD)^b^ | 7.65 (4.81) | 6.77 (4.34) | 5.89 (4.57) | 4.71 (4.26) |
| Follow-up duration (year)^ab^ | 1.84 (1.05) | 2.44 (0.90) | 1.93 (1.13) | 2.41 (1.00) |
| CDR-SB^b^ | 0.32 (0.28) | 0.30 (0.25) | 1.73 (0.78) | 1.93 (0.86) |

*Note.* ^a^ CU-Unconvert ≠ CU-Convert; ^b^ MCI-Unconvert ≠ MCI-Convert. *Abbreviations.* CU: Cognitively unimpaired; MCI: Mild cognitive impairment; SD: Standard deviation. CDR-SB: Clinical Dementia Rating-Sum of Boxes.

**Supplementary Table 2.** Demographic and clinical characteristics of individuals with suspected AD and vascular etiologies

|  | AD | Vascular origin | Statistical comparison |
| --- | --- | --- | --- |
| N | 933 | 300 |  |
| Age (years, mean [SD]) | 74.83 (8.66) | 72.50 (8.83) | *F* _1, 1231_ = 16.25, *p* < 0.001 |
| Gender (% female [n]) | 62.27 (581/933) | 41.66 (125/300) | *χ*^2^ *_df_* _= 1,_ *_n_* _= 1233_ = 39.38, *p* < 0.001 |
| Education (years, mean [SD]) | 5.37 (4.49) | 5.99 (4.68) | *F* _1, 1231_ = 4.25, *p* < 0.05 |
| CDR-SB (mean [SD]) | 1.07 (1.07) | 1.10 (1.06) | *F* _1, 1231_ = 6.20, *p* < 0.05 |
| Conversion time (years, mean [SD]) | 2.64 (1.34) | 2.55 (1.27) | *F* _1, 1231_ = 1.04, *p* = 0.31 |

*Abbreviations.* AD: Alzheimer’s disease.

**Supplementary Table ~~2~~3.** AUC and cutoff points of CASI between individuals with CU and dementia

| Group | AUC | 95% CI | Cutoff | Sensitivity | Specificity |
| --- | --- | --- | --- | --- | --- |
| *CU vs. Dementia* |  |  |  |  |  |
| Age |  |  |  |  |  |
| < 75 | 0.95 | 0.94-0.96 | 74/75 | 0.90 | 0.87 |
| ≥ 75 | 0.95 | 0.94-0.96 | 60/61 | 0.91 | 0.86 |
| Education |  |  |  |  |  |
| 0 | 0.96 | 0.94-0.97 | 50/51 | 0.95 | 0.85 |
| < 6 | 0.94 | 0.92-0.96 | 61/62 | 0.89 | 0.87 |
| ≥ 6 | 0.96 | 0.96-0.97 | 74/75 | 0.92 | 0.89 |
| Younger-illiterate | 0.94 | 0.90-0.97 | 58/59 | 0.92 | 0.84 |
| Younger-low education | 0.92 | 0.89-0.96 | 67/68 | 0.86 | 0.89 |
| Younger-high education | 0.95 | 0.94-0.96 | 77/78 | 0.90 | 0.88 |
| Older-illiterate | 0.96 | 0.94-0.97 | 50/51 | 0.94 | 0.86 |
| Older-low education | 0.94 | 0.91-0.96 | 59/60 | 0.89 | 0.86 |
| Older-high education | 0.96 | 0.95-0.97 | 72/73 | 0.88 | 0.91 |

*Note.* Younger: < 75 years; Older: ≥ 75 years; Low education: < 6 years; High education: ≥ 6 years. All *p* values < .001. *Abbreviations.* AUC: Area under the curve; CASI: Cognitive assessment screening instrument; CI: Confidence interval.

**
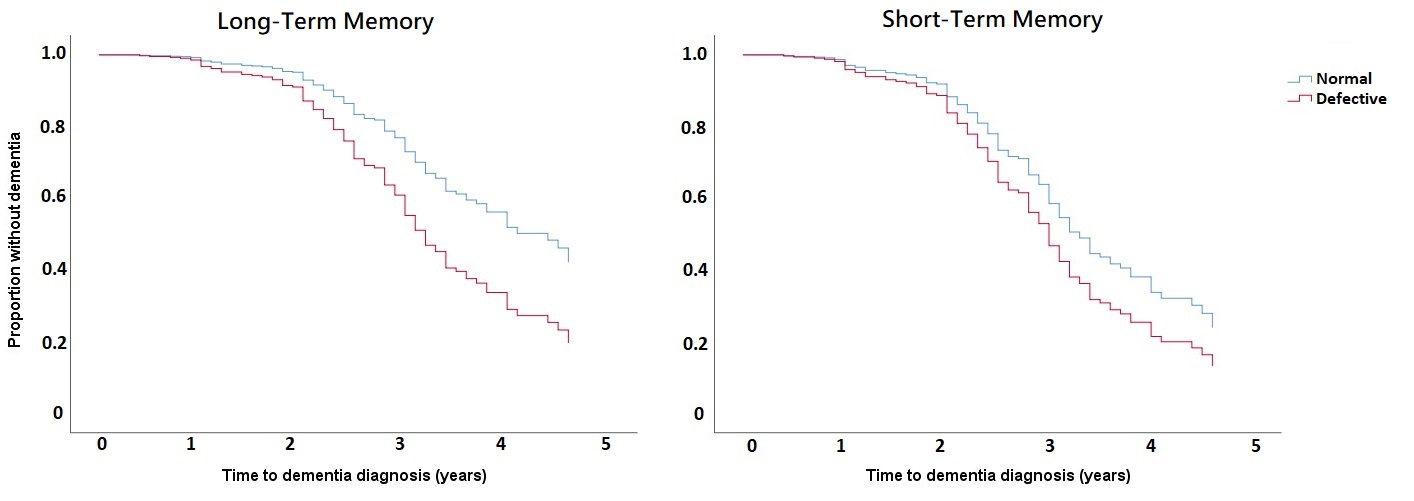
**

**Supplementary Fig. 1.** Survival curves of dementia between individuals with poor performance on memory tasks of CASI.

**
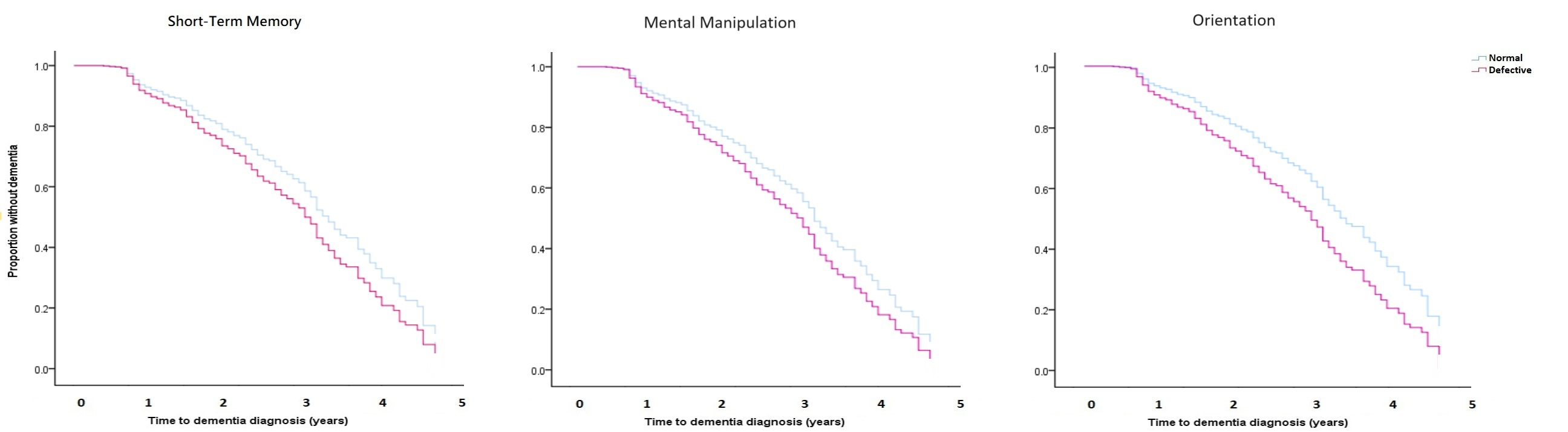
**

**Supplementary Fig. 2.** Survival curves of DAT between individuals with poor performance on memory tasks of CASI.

**
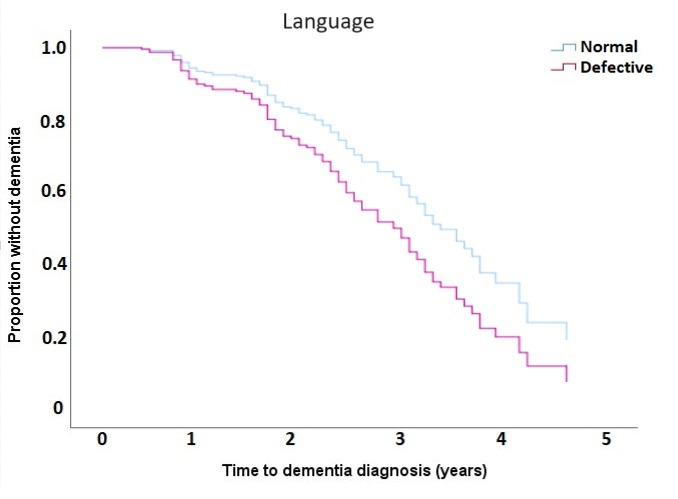
**

**Supplementary Fig. 3.** Survival curves of VaD between individuals with poor performance on memory tasks of CASI.
